# Decipher the spatial dynamics of the cell state transition and lineage development during cancer evolution

**DOI:** 10.1093/bib/bbaf631.006

**Published:** 2025-12-12

**Authors:** LI Jiabao, Jiguang Wang

**Affiliations:** Division of Life Science, Department of Chemical and Biological Engineering, and State Key Laboratory of Nervous System Disorders, The Hong Kong University of Science and Technology, Hong Kong SAR, China; Division of Life Science, Department of Chemical and Biological Engineering, and State Key Laboratory of Nervous System Disorders, The Hong Kong University of Science and Technology, Hong Kong SAR, China

## Abstract

**Background:**

Advances in single-cell and spatial transcriptomics have transformed our understanding of tumor cell states and lineage dynamics, enabling high-resolution investigation of cellular heterogeneity and evolution.

**Motivation:**

Building on Schiffman et al.’s Markovian framework for inferring cell state transitions from single-cell lineage tracing, there is a need for models that integrate spatial context to better capture the dynamics of tumor evolution.

**Method and results:**

We introduce StateSim, a spatial–temporal model that simulates tumor cell state transitions within tissue, incorporating spatial growth and mutation accumulation (Waclaw, et al. 2015). StateSim assumes that daughter cells are spatial neighbors, shaping the spatial organization of cell states, and models two cell states with distinct birth and death rates. To quantify spatial patterns, we developed StateMap, a network-based approach that maps tumor cell states and their spatial relationships, summarizing transition patterns through network statistics. Applying StateSim under various transition scenarios, we demonstrate that distinct transition modes yield unique spatial statistics, robustly correlated with transition rates (Pearson *r* = 0.9938, *P* = 6.87e-10). Using spatial transcriptomics data from glioma samples (Greenwald, et al. 2024), StateMap identified subgroups with distinct transition patterns, further validated by spatial statistics such as bivariate Moran’s I. Approximate Bayesian computation with StateSim enabled inference of posterior probabilities for cell state transition rates and phylogenetic trajectories.

**Conclusion:**

Our framework enables inference of cell state transitions and in-silico lineage tracing from spatial data, advancing studies of tumor heterogeneity and evolution.

**Significance:**

This work provides a novel computational approach for integrating spatial and temporal information to decipher the dynamics of cell state transitions and lineage development during cancer evolution, offering new insights into tumor heterogeneity and progression.

**References:**

1. Schiffman J.S., et al. ‘Defining heritability, plasticity, and transition dynamics of cellular phenotypes in somatic evolution.’ Nature Genetics 2024; 56(10):2174–2184.

2. Waclaw B., et al. ‘A spatial model predicts that dispersal and cell turnover limit intratumour heterogeneity.’ Nature 2015; 525(7568):261–264.

3. Greenwald A.C., et al. ‘Integrative spatial analysis reveals a multi-layered organization of glioblastoma.’ Cell 2024; 187(10):2485–2501.

